# Deciphering the Relationships Between Soil Enzymatic Activities and N- and P-Cycling Functional Genes Under Long-Term Fertilization

**DOI:** 10.3390/microorganisms13122719

**Published:** 2025-11-28

**Authors:** Dong Xue, Shumiao Jiang, Na Zhao, Mengnan Yao, Enqiang Zhou, Yongqiang Wang, Furong Dong, Xue Gao, Xuejun Wang, Yamei Miao, Libin Wei, Kaihua Wang, Anyong Hu

**Affiliations:** 1 Jiangsu Yanjiang Institute of Agricultural Sciences, Nantong 226541, China; xuedongjsrg@jaas.ac.cn (D.X.); zhaona5670@163.com (N.Z.); 20212004@jaas.ac.cn (M.Y.); zhouenqiang0526@163.com (E.Z.); 20162001@jaas.ac.cn (Y.W.); wangxj4002@sina.com (X.W.); 19972005@jaas.ac.cn (Y.M.); 20212013@jaas.ac.cn (L.W.); 2 School of Geographic Science, Nantong University, Nantong 226019, China; jiangshumiao66@163.com; 3 Qidong Agricultural Technology Extension Center, Nantong 226200, China; qdzzz@126.com (F.D.); aphidm@126.com (X.G.)

**Keywords:** long-term fertilization, soil fertility, soil enzyme activity, nitrogen cycling, phosphorus cycling, functional genes

## Abstract

Long-term fertilization profoundly influences soil biochemical processes and microbial functionality, yet the coupling mechanisms between soil enzyme activities and functional genes in nutrient cycling remain unclear. This study investigated the effects of different fertilization regimes—nitrogen alone (N), nitrogen–phosphorus–potassium fertilizer (NPK), organic fertilizer (M), and combined organic–inorganic fertilizer (MNPK)—on soil properties, enzyme activities, N- and P-cycling-related functional gene abundances, and faba bean (*Vicia faba* L.) yield in a 45-year ongoing field experiment in subtropical eastern China. Results showed that long-term fertilization significantly affected soil pH, electrical conductivity, nutrient contents, and crop yield. Organic fertilizer addition (M and MNPK) markedly improved soil organic matter, total and available nutrients, and enhanced faba bean grain yield by 75.07–92.79% compared with NPK, whereas NPK had limited benefits on total and available soil nutrients compared with N-only application. Soil enzyme activity analysis revealed that the MNPK treatment achieved the highest urease and neutral protease activities, while acid and alkaline protease activities responded inconsistently. Phosphorus-related enzymes (acid, neutral, and alkaline phosphatases) were strongly stimulated by organic inputs, reflecting enhanced P mineralization potential. Functional gene analysis showed that N-fixation and assimilatory nitrate reduction genes increased under M and MNPK, while N assimilation, N mineralization, anammox, nitrification, denitrification, and dissimilatory nitrate reduction genes were enriched under N treatment. Phosphate uptake and transport genes were upregulated under NPK, M, and MNPK, whereas inorganic P solubilization genes were highest under N. Significant positive correlations were observed among soil enzyme activities, nutrient contents, and faba bean yield, whereas acid and alkaline protease activities showed opposite trends. The relative abundances of N- and P-cycling functional genes exhibited distinct yet coordinated relationships with soil fertility indicators and enzyme activities. These findings provide mechanistic insights into the long-term regulation of soil–microbe interactions and nutrient cycling, offering a scientific basis for sustainable fertilization strategies in agroecosystems.

## 1. Introduction

Soil enzymes constitute indispensable and dynamic components within the soil ecosystem, originating from soil microorganisms, plant roots, soil fauna, and plant residues [1,2,3]. They play a pivotal role in the mineralization and degradation of soil organic substrates, thereby driving nutrient mobilization and transformation [4,5]. They effectively regulate the kinetics of soil nutrient cycles [6], exerting a significant influence on soil fertility and crop productivity. Soil enzyme activity serves as a critical indicator for assessing soil fertility, reflecting the intensity and temporal dynamics of various nutrient metabolic processes in soils [7,8]. Compared with soil physicochemical properties, soil enzyme activity is highly dynamic and particularly sensitive to environmental management practices. Notably, agronomic interventions such as tillage regimes [9,10], crop rotation systems [11,12], and fertilization strategies [13,14] have been shown to strongly influence soil enzyme activity.

Enhancing soil fertility represents a fundamental pathway for increasing crop growth potential, yield, and achieving high nutritional value [15]. While fertilization serves as the primary measure for improving soil fertility, long-term indiscriminate application of chemical fertilizers can diminish soil fertility. Such yield-oriented fertilization strategies, pursued at environmental cost, exert detrimental effects on soil enzyme activities [16,17]. Long-term field experiments have consistently demonstrated that continuous application of N, P, and K fertilizers suppresses soil enzyme activity [18,19]. Conversely, rational fertilization strategies can effectively enhance soil enzyme activity, thereby promoting soil metabolism processes, altering soil nutrient forms, increasing soil fertility, and improving soil properties [20,21,22]. Consequently, exploring optimal fertilization management practices is particularly important.

Organic fertilizers, as mature amendments, substantially increase soil organic matter (SOM) content when incorporated into soil. This not only improves soil physicochemical properties but also provides favorable environmental conditions and energy substrates for microbial growth [23,24,25]. SOM, a key determinant of soil enzyme activities, is decomposed by microorganisms into a diverse range of products that supply essential nutrients and energy to both microbes and plants, and induce the production of soil urease, phosphatase, and protease, thereby modulating enzymatic activity [26,27,28]. Long-term field experiments conducted by Zhang et al. [1] revealed that sustained manure application elevated activities of soil enzymes such as acid phosphatase and significantly raised soil total P content. Similarly, in a 12-year tobacco-maize rotation trial, Jiang et al. [29] found that adding organic fertilizer increased crop yields, soil acid phosphatase activity, and overall soil nutrient status. A five-year study on winter wheat likewise verified that organic fertilizer application enhanced the activities of soil urease and protease, with crop yield and soil organic carbon being significantly positively correlated with urease and protease activities [30]. Adequate soil organic nutrient pools are generally associated with elevated soil enzyme activity, thereby accelerating SOM mineralization rates and boosting microbial metabolic vigor. Moreover, the chemical decomposition of SOM is a continuous, long-term process that helps maintain a dynamic balance of nutrient supply, thereby enhancing the ecological sustainability of soil nutrient cycling [31,32].

Soil enzymes play an important role in plant nutrient cycling and utilization efficiency [33]. However, the extent of their activities ultimately depends on the metabolic potential of the soil microbial community [22]. Functional genes, as the molecular basis of microbial metabolic functions, directly regulate enzyme synthesis and expression, and their distribution and expression profiles of these functional genes are strongly shaped by soil environmental conditions [34,35,36]. Long-term fertilization, by altering soil physicochemical properties, microbial community composition and structure, can directly or indirectly regulate the relative abundances of functional genes involved in N and P cycling [37,38,39]. For instance, long-term application of chemical fertilizers alone may reduce the abundance of N-fixation functional genes and organic-phosphorus mineralization functional genes as a consequence of soil acidification and nutrient imbalance [40,41], whereas organic fertilizer inputs can increase the abundance of ammonia-oxidizing and phosphatase functional genes by enhancing carbon source availability and improving soil environment [42,43]. Elucidating the mechanistic linkages between shifts in functional gene abundance and corresponding changes in soil enzyme activity is essential for revealing the underlying pathways through which fertilization practices regulate soil nutrient cycling [44]. Meanwhile, soil enzymes, which mediate SOM decomposition and nutrient mineralization, have been used as sensitive bioindicators for assessing the effectiveness of agricultural management practices [45].

Although many studies have examined the effects of long-term fertilization on soil microbial communities or enzyme activities separately, few have systematically integrated multi-enzyme activities with the functional gene profiles involved in N- and P-cycling to elucidate their coordinated regulatory mechanisms. Moreover, the quantitative relationships among soil chemical properties, enzyme activities, and microbial functional potentials under contrasting fertilization regimes remain largely unexplored, particularly in multi-decadal field systems. Therefore, this study aimed to fill these knowledge gaps by investigating the relationships between soil enzyme activities and the relative abundances of N- and P-cycling functional genes under long-term fertilization in a 45-year ongoing field experiment. Specifically, we sought to address three key questions: (1) how soil enzyme activities respond to different long-term fertilization treatments; (2) how microbial functional genes encoding nitrogen- and phosphorus-cycling enzymes change under these treatments; and (3) how soil enzyme activities are correlated with the relative abundances of these functional genes, soil properties, and faba bean yield.

## 2. Materials and Methods

### 2.1. Experimental Site

The long-term field trial was located at the experimental base of the Jiangsu Yanjiang Institute of Agricultural Sciences in Xueyao Town, Rugao City, Jiangsu Province, China (120°37′ E, 32°07′ N). This area experiences a subtropical monsoon climate, characterized by mild temperatures (mean annual temperature of 16.2 °C) and abundant rainfall averaging 1250 mm per year. The soil at the site is a fluvo-aquic type developed from alluvial deposits of the Yangtze River and exhibits a sandy-loam texture. When the experiment was initiated in 1979, the topsoil (0–15 cm) contained 14.4 g kg^−1^ of soil organic matter, 133 mg kg^−1^ of alkali-hydrolyzable nitrogen, 29.0 mg kg^−1^ of available phosphorus, 64.0 mg kg^−1^ of available potassium, and had a pH of 7.86.

### 2.2. Experimental Design

The long-term experiment has been continuously maintained since 1979 under a six-season crop rotation system within a three-year cycle: rice (*Oryza sativa* L.), faba bean (*Vicia faba* L.), maize (*Zea mays* L.), barley (*Hordeum vulgare* L.), cotton (*Gossypium* spp.), and wheat (*Triticum aestivum* L.). In June 2022, after the wheat harvest, the crop rotation system was adjusted to wheat–soybean–faba bean–maize–barley–rice, while the long-term fertilization treatments and management regimes were maintained consistently. The current study was based on soil and plant samples collected in May 2023 from this ongoing long-term fertilization trial.

The experiment included four fertilization treatments: nitrogen alone (N), NPK fertilizer (NPK), organic fertilizer alone (M), and NPK plus organic fertilizer (MNPK). A randomized complete block design was used, with a plot area of 16.8 m^2^ and four replicates. For faba bean, 45 kg N ha^−1^ was applied as urea, while for other crops a rate of 225 kg N ha^−1^ was used. All crops received 24.56 kg P ha^−1^ as superphosphate and 139.00 kg K ha^−1^ as potassium chloride. In the M and MNPK treatments, organic fertilizer (pig manure) was applied at 18 t ha^−1^. The pig manure had a pH of 7.6, organic matter 312.2 g kg^−1^, N 5.7 g kg^−1^, P 3.0 g kg^−1^, and K 3.6 g kg^−1^. Phosphorus, potassium, and pig manure were supplied before planting as base fertilization. Nitrogen was applied as topdressing for faba bean, and split (40% basal, 60% topdressing) for the other crops. At grain maturity, all crops were manually harvested, and no straw was returned to the field.

### 2.3. Soil Sampling

Soil sampling was conducted on 4 May 2023 during the maturation stage of faba bean. In each plot, five soil cores (0–20 cm) were collected using a hollow auger and subsequently mixed thoroughly to produce one representative composite sample. The collected soils were immediately sealed in sterile bags and transported to the laboratory in insulated containers packed with ice. Upon arrival, the fresh soil was gently sieved through a 2 mm mesh to eliminate fine roots and surface debris. A portion of the fresh soil was directly used for measuring soil moisture and for extracting inorganic nitrogen. Approximately 50 g of fresh soil from each sample was frozen at −80 °C for DNA analysis. The remaining soil was air-dried in a shaded environment, finely ground, and sieved through 0.85 mm and 0.149 mm meshes for subsequent chemical determinations.

### 2.4. The Determinations of Soil Properties, Crop Yield, and Soil Enzyme Activities

The measured soil indicators included pH, electrical conductivity (EC), soil organic matter (SOM), total carbon (TC), total nitrogen (TN), total phosphorus (TP), ammonium-N (NH_4_^+^-N), nitrate-N (NO_3_^−^-N), available phosphorus (AP), and available potassium (AK). Soil pH and EC were measured using soil suspensions prepared with water at ratios of 1:2.5 (*w*/*v*) and 1:5 (*w*/*v*), respectively, and analyzed using a pH meter and a portable conductivity meter (Mettler-Toledo, Shanghai, China). SOM was quantified following the dichromate oxidation procedure specified in Chinese Standard GB9834-88 [46]. Total carbon and nitrogen were determined by high-temperature combustion method with an elemental analyzer (Vario EL CUBE, Elementar, Langenselbold, Germany). TP was analyzed after acid digestion using the molybdenum–antimony colorimetric method. For NH_4_^+^-N and NO_3_^−^-N, 5 g of fresh soil was shaken for 1 h with 1 M KCl (soil:solution = 1:5), after which the filtrates were quantified using a continuous-flow analyzer (San++, Skalar, Netherlands). Available P was extracted with 0.5 M NaHCO_3_ by shaking at 180 rpm for 30 min and determined by UV spectrophotometry based on the molybdenum blue method. AK was extracted with 1 M ammonium acetate by shaking at 120 rpm for 30 min and measured with a flame photometer. At the faba bean maturity stage, whole plants from each plot were manually harvested, air dried, and then threshed to determine grain yield.The activities of soil urease, acid protease, neutral protease, alkaline protease, acid phosphatase, neutral phosphatase, and alkaline phosphatase were quantified with soil enzyme assay kits (Solarbio Science & Technology Co., Beijing, China). For urease, one activity unit corresponded to the enzymatic generation of 1 μg NH_3_-N per g of soil per day. Soil protease activity (acidic, neutral, or alkaline) was expressed as the amount of enzyme producing 1 μmol tyrosine per gram soil per day. One unit of acid, neutral, or alkaline phosphatase activity was defined as the amount of enzyme releasing 1 nmol phenol per g of soil per day at 37 °C.

### 2.5. Functional Gene Screening

Based on the 16S rRNA gene amplicon sequencing data, the functional potential of soil bacterial communities was predicted using Tax4Fun2 [47]. It should be noted that Tax4Fun2 provides inferred rather than directly measured functional profiles, representing the potential functional capacity of microbial communities rather than actual gene expression. Functional annotations in Tax4Fun2 rely on the Kyoto Encyclopedia of Genes and Genomes (KEGG) database, and the predictions are reported as relative abundances. Following previous studies [44,48,49], a total of 151 KEGG functional pathways were selected and categorized into 14 metabolic pathways associated with N and P cycling. Details are provided in Table A1.

### 2.6. Statistical Analysis

Data analysis was carried out using Microsoft Excel, with results expressed as mean ± standard deviation. Differences among treatments were evaluated using one-way ANOVA in SPSS 27.0, followed by Duncan’s multiple range test to determine statistically significant variations. Pearson correlation analysis was conducted in R (4.3.1) via the cor package to examine the relationships among soil properties, faba bean yield, the relative abundances of N- and P-cycling functional genes, and soil enzyme activities. The correlation results were visualized with the corrplot package, while bar charts and boxplots were generated using Origin 2025.

## 3. Results

### 3.1. The Effects of Long-Term Fertilization Regimes on Soil Properties and Faba Bean Yields

Table 1 demonstrates that long-term fertilization regimes significantly influenced soil properties and faba bean yield. Compared with sole N or organic fertilizer application, NPK and MNPK treatments markedly decreased soil pH, with the lowest value under MNPK. MNPK also significantly increased soil EC, while no significant variation was detected among the remaining treatments. The use of organic fertilizer, either applied independently (M) or jointly with mineral fertilizers (MNPK), substantially enhanced soil fertility indices, including SOM, TC, TN, TP, AP, and AK, compared with chemical fertilizer treatments. In contrast, balanced NPK fertilization provided only marginal improvements over sole N, without statistical significance. No significant treatment effects were observed for NO_3_^−^-N or NH_4_^+^-N contents. Faba bean yield increased by 1097.32% under NPK compared with sole N, and was further promoted by organic fertilizer treatments, with M and MNPK increasing grain yield by 75.07% and 92.79% over NPK, respectively, although the difference between M and MNPK was not significant.

### 3.2. Effects of Long-Term Fertilization Treatments on Soil N-Related Enzyme Activities

**Figure 1:**
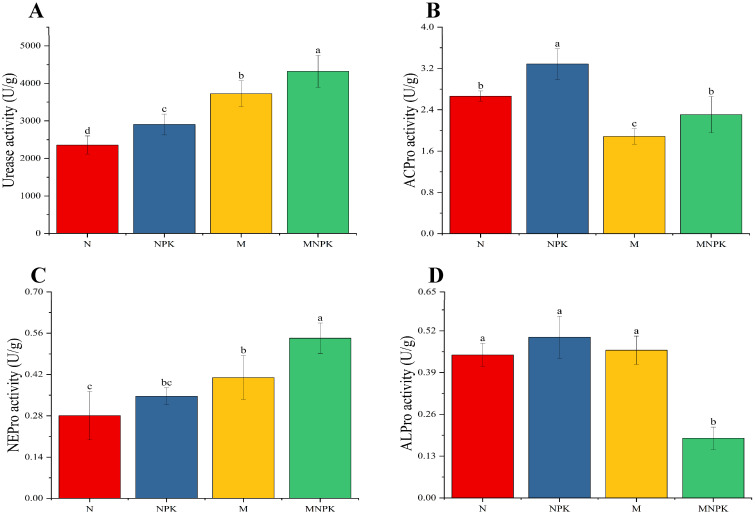
Activity of soil N-related enzymes under long-term fertilization treatments. (**A**) Soil urease (Urease); (**B**) Soil acid protease (ACPro); (**C**) Soil neutral protease (NEPro); (**D**) Soil alkaline protease (ALPro). Error bars represent standard deviations, and different lowercase letters above the bars indicate significant differences among treatments at *p* < 0.05.

As shown in Figure 1, compared with sole N application, all other fertilization treatments significantly increased soil urease activity to varying extents (Figure 1A), following the order of MNPK > M > NPK > N, with respective increases of 83.21%, 58.04%, and 23.24%. The mean activities of soil acid protease, neutral protease, and alkaline protease under long-term fertilization treatments were 2.5354 U/g, 0.3951 U/g, and 0.3976 U/g, respectively (Figure 1B–D). Specifically, compared with N-only treatment, NPK significantly enhanced soil acid protease activity by 23.26%. However, acid protease activity under both M and MNPK treatments was significantly lower than under NPK, with no significant difference observed between N and MNPK (Figure 1B). For soil neutral protease activity, no significant differences were observed between N and NPK treatments, whereas organic fertilizer application significantly increased neutral protease activity, with the highest activity under MNPK (Figure 1C). In contrast, soil alkaline protease activity showed no significant differences among N, NPK, and M treatments, but was significantly reduced under the MNPK treatment (Figure 1D).

### 3.3. Effects of Long-Term Fertilization Regimes on Soil P-Related Enzyme Activities

**Figure 2:**
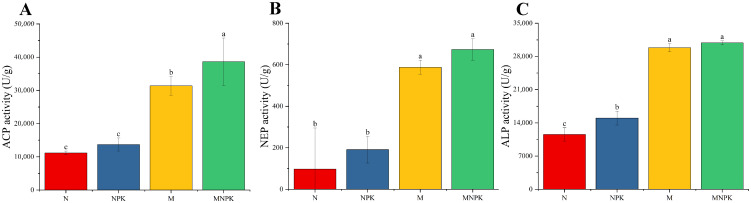
Activity of soil P-related enzymes under long-term fertilization treatments. (**A**) Soil acid phosphatase (ACP); (**B**) Soil neutral phosphatase (NEP); (**C**) Soil alkaline phosphatase (ALP). Different lowercase letters above the error bars indicate statistically significant differences in enzyme activities among treatments (*p* < 0.05).

Under long-term fertilization regimes, soil acid, neutral, and alkaline phosphatase activities exhibited similar variation patterns (Figure 2). Application of organic fertilizer alone or in combination with inorganic fertilizers significantly enhanced the activities of acid, neutral, and alkaline phosphatases compared with chemical fertilizer treatments, corresponding to 1.81~2.46-fold, 5.01~5.89-fold, and 1.58~1.67-fold increases relative to N and NPK treatments, respectively. Except for alkaline phosphatase, where NPK treatment showed significantly higher activity than N treatment, no significant differences were found between N and NPK treatments for either acid or neutral phosphatase activities (Figure 2B).

### 3.4. Effects of Long-Term Fertilization Regimes on the Functional Diversity of Soil Bacterial Communities

**Figure 3:**
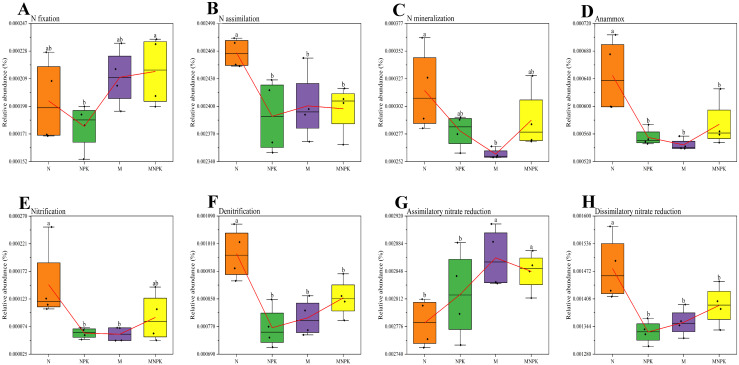
Enzyme-encoding genes related to nitrogen cycling in soil bacterial communities under long-term fertilization regimes. (**A**) N fixation; (**B**) N assimilation; (**C**) N mineralization; (**D**) Anammox; (**E**) Nitrification; (**F**) Denitrification; (**G**) Assimilatory nitrate reduction; (**H**) Dissimilatory nitrate reduction. Red lines represent mean values. Different lowercase letters above the error bars indicate significant differences in soil enzyme activity among treatments (*p* < 0.05, Duncan’s test).

The relative abundances of N-cycling functional genes exhibited distinct response patterns under the four long-term fertilization treatments (Figure 3). Among them, the abundance of N fixation genes was highest under MNPK, significantly exceeding that under NPK, but no significant differences were observed compared with N or M (Figure 3A). Genes associated with N mineralization showed the highest abundance under N treatment and the lowest under M, while NPK and MNPK treatments did not differ significantly from N (Figure 3C). The N-only treatment markedly increased the abundance of nitrification genes, with values significantly higher than under NPK and M. Although MNPK was also lower than N, the difference between MNPK and N was not statistically significant (Figure 3E). Organic fertilizer application significantly enhanced the abundance of assimilatory nitrate reduction genes compared with chemical fertilizer treatments; however, no significant differences were detected between M and MNPK or between N and NPK (Figure 3G). Functional genes related to N assimilation, anammox, denitrification, and dissimilatory nitrate reduction displayed similar patterns, with the highest abundances under N treatment, significantly exceeding those of the other treatments, while NPK, M, and MNPK showed no significant differences (Figure 3B,D,F,H).

**Figure 4:**
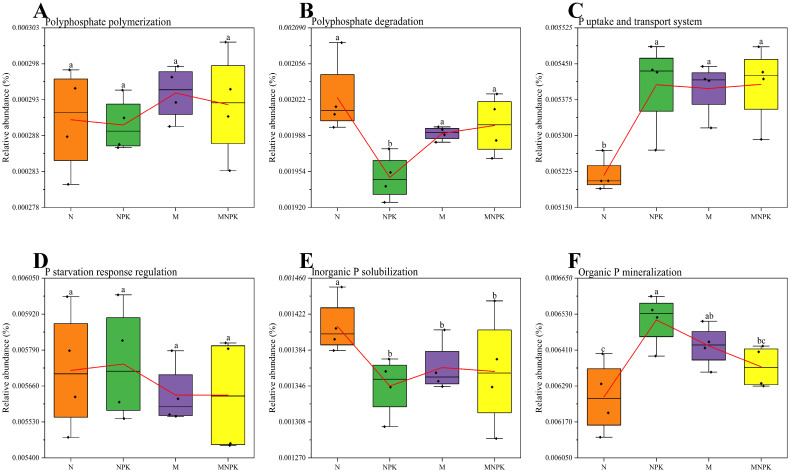
Enzyme-encoding genes related to P-cycling in soil bacterial communities under long-term fertilization treatments. (**A**) Polyphosphate polymerization; (**B**) Polyphosphate degradation; (**C**) P uptake and transport; (**D**) P starvation response regulation; (**E**) Inorganic P solubilization; (**F**) Organic P mineralization. The red connecting lines represent mean values across replicates, and different lowercase letters above the short horizontal bars denote significant differences in gene abundances among treatments (*p* < 0.05, Duncan’s test).

Among the enzyme-coding genes related to the P-cycling (Figure 4), the relative abundances of genes associated with polyphosphate polymerization and P starvation response regulation exhibited no significant differences across different treatments (Figure 4A,D). The relative abundance of genes involved in polyphosphate degradation was highest under the N, M, and MNPK treatments than that under the balanced NPK fertilization (Figure 4B). Genes related to phosphate uptake and transport exhibited the lowest relative abundance under the N treatment, significantly lower than those under NPK, M, and MNPK treatments, whereas no significant differences were observed among the latter three treatments (Figure 4C). Compared with the N treatment, the relative abundances of inorganic P solubilizing genes were markedly lower in the NPK, M, and MNPK treatments, while no significant differences were found among these three treatments (Figure 4E). The relative abundance of genes involved in organic P mineralization was highest in the NPK treatment, showing significant differences compared with the N and MNPK treatments, but not with the M treatment (Figure 4F).

### 3.5. Correlation Analysis of Soil Properties and Faba Bean Yield with Soil Enzyme Activities

**Figure 5:**
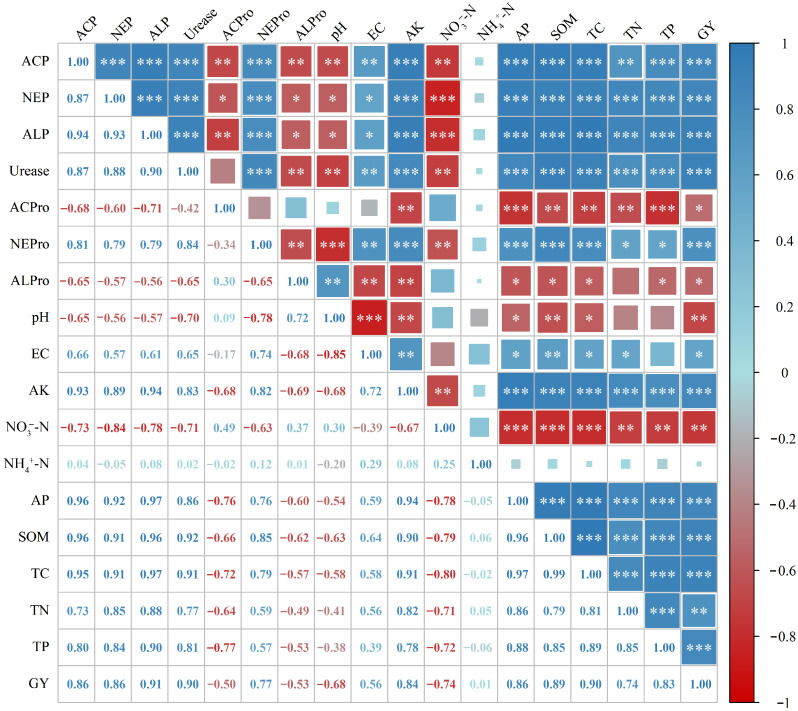
Correlation analysis of soil properties and faba bean yield with soil enzyme activities. Pearson correlation coefficient, * *p* < 0.05; ** *p* < 0.01; *** *p* < 0.001.

This study revealed the correlation patterns among soil properties, soil enzyme activities, and faba bean yield under long-term fertilization regimes through correlation analysis (Figure 5). The results showed that, under different long-term fertilization treatments, the activities of soil acid phosphatase, neutral phosphatase, alkaline phosphatase, urease, and neutral protease were all highly positively correlated with each other. The activity of soil acid protease showed a significant negative relationship with soil acid phosphatase, neutral phosphatase, and alkaline phosphatase activities, whereas soil alkaline protease activity was significantly negatively correlated with soil acid phosphatase, neutral phosphatase, alkaline phosphatase, urease, and neutral protease activities.

Significant correlations were also observed between soil enzyme activities and various soil properties. The activities of soil acid phosphatase, neutral phosphatase, alkaline phosphatase, urease, and neutral protease showed consistent trends of correlation with soil properties and faba bean yield, being significantly or highly positively correlated with EC, AK, AP, SOM, TC, TN, TP, and GY, while significantly negatively correlated with pH and NO_3_^−^-N, and showing no significant correlation with NH_4_^+^-N. In contrast, the activities of soil acid protease and alkaline protease generally exhibited opposite trends, being significantly negatively correlated with EC, AK, AP, SOM, TC, TN, TP, and GY (except for soil acid protease vs. EC and soil alkaline protease vs. TN, which showed no significant correlations). Soil alkaline protease activity was significantly positively correlated with pH, whereas soil acid protease activity showed no significant correlation with pH, and both soil acid and alkaline protease activities were not significantly correlated with NH_4_^+^-N and NO_3_^−^-N.

### 3.6. Correlation Analysis Between Soil Properties and Faba Bean Yield with the Relative Abundance of Soil N- and P-Related Functional Genes

**Figure 6:**
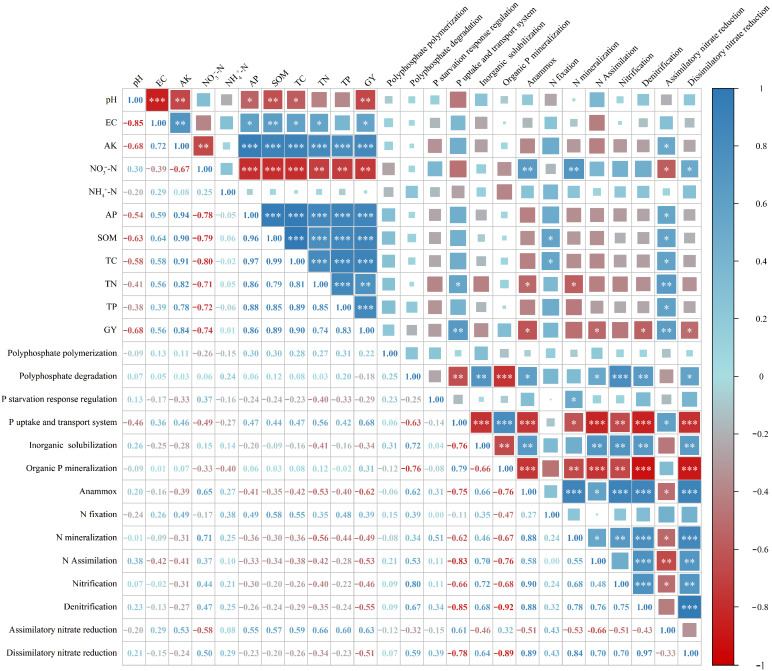
Correlation analysis of soil properties and faba bean grain yield with the relative abundances of N- and P-cycling functional genes. Note: Correlation analysis (Pearson correlation coefficient, * *p* < 0.05; ** *p* < 0.01; *** *p* < 0.001) revealed distinct functional partitioning in the associations between soil properties, faba bean grain yield (GY), and the abundances of N- and P-cycling functional genes.

Correlation analysis (Figure 6) revealed distinct functional partitioning in the associations between soil properties, faba bean grain yield (GY), and the abundances of N- and P-cycling functional genes.

Regarding soil P-cycling genes, only genes related to the phosphate uptake and transport system were significantly correlated with TN and GY, whereas other P-cycling functional genes showed weak and non-significant correlations with soil properties and GY. For N-cycling genes, anammox genes were positively correlated with NO_3_^−^-N but significantly negatively correlated with TN and GY. N fixation genes were positively correlated with SOM and TC. N mineralization genes were positively correlated with NO_3_^−^-N but negatively correlated with TN. N assimilation and denitrification genes were all significantly negatively correlated with GY. Assimilatory nitrate reduction genes showed significant positive correlations with SOM, TC, TN, TP, AP, AK, and GY, but were negatively correlated with NO_3_^−^-N. In contrast, dissimilatory nitrate reduction genes were significantly positively correlated with NO_3_^−^-N and negatively correlated with GY. Soil pH, EC, and NH_4_^+^-N were not significantly correlated with any N- and P-cycling functional genes.

### 3.7. Correlation Analysis Between Soil Enzyme Activities and the Relative Abundance of Soil N- and P-Related Functional Genes

**Figure 7:**
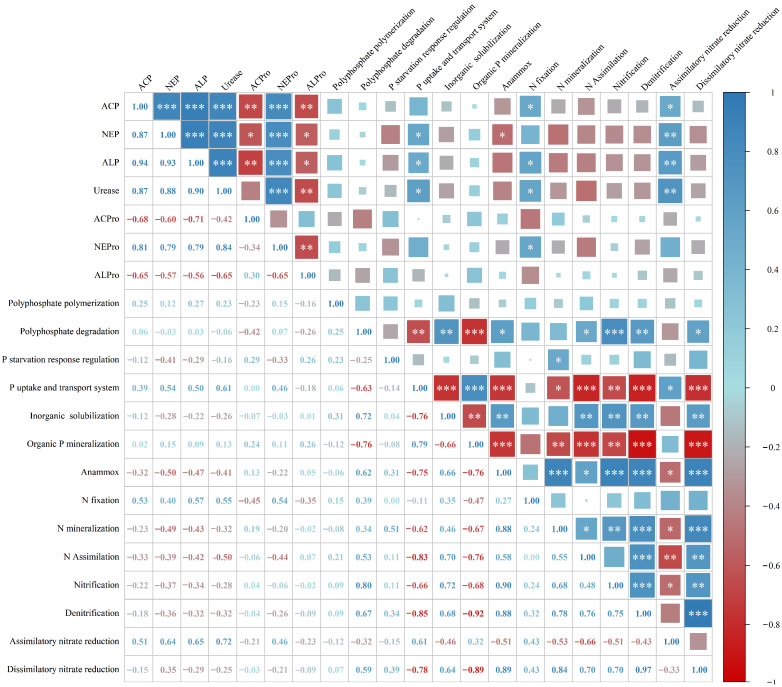
Correlation analysis between soil enzyme activities and the relative abundance of N- and P-related functional genes. Pearson correlation coefficient, * *p* < 0.05; ** *p* < 0.01; *** *p* < 0.001.

Correlation analysis (Figure 7) indicated that soil neutral phosphatase, alkaline phosphatase, and urease activities were significantly positively correlated with phosphate uptake and transport functional genes. Anammox genes were negatively correlated with neutral phosphatase activity. N fixation genes showed significant positive correlations with soil acid phosphatase, alkaline phosphatase, urease, and neutral protease activities. Assimilatory nitrate reduction genes were significantly positively correlated with soil acid phosphatase, neutral phosphatase, alkaline phosphatase, and urease activities. Moreover, N- and P-cycling functional genes exhibited clear synergistic and antagonistic relationships.

Polyphosphate polymerization genes were not significantly correlated with any N-cycling functional genes. In contrast, polyphosphate degradation genes were significantly positively correlated with anammox, nitrogen assimilation, nitrification, denitrification, and dissimilatory nitrate reduction genes. P starvation response regulation genes showed significant positive correlations with N mineralization genes. Phosphate uptake and transport system genes exhibited strong negative correlations with multiple N transformation pathways, including anammox, nitrogen mineralization, nitrogen assimilation, nitrification, denitrification, and dissimilatory nitrate reduction. In addition, phosphate uptake and transport functional genes were significantly positively correlated with assimilatory nitrate reduction genes.

Similarly, organic P mineralization genes were significantly negatively correlated with anammox, N mineralization, N assimilation, nitrification, denitrification, and dissimilatory nitrate reduction pathways. In contrast, inorganic P solubilization genes were generally significantly positively correlated with these N transformation genes, including anammox, N assimilation, nitrification, denitrification, and dissimilatory nitrate reduction, showing consistent directional patterns.

## 4. Discussion

### 4.1. Relationships Between Long-Term Fertilization Regimes and N- and P-Related Soil Enzyme Activities

The primary function of soil urease is to hydrolyze urea into carbon dioxide and ammonia [50], and its activity can reflect the soil nitrogen supply level [51]. Soil urease activity is regulated by multiple environmental factors, such as soil texture, land use type, soil organic carbon content, and total nitrogen content [52,53,54]. Under the sole N treatment, the soil is simultaneously constrained by insufficient carbon sources and limitations of P and K, resulting in the lowest soil urease activity. In contrast, NPK application markedly promotes faba bean root growth and biomass, thereby supplying microbes with a certain amount of carbon via root exudates [55,56]. The M and MNPK treatments further increase carbon supply and nutrient availability to microbes, leading to the highest soil urease activity [57,58,59], which is consistent with the observed significant positive correlations between soil urease activity and SOM, TC, and both total and available nutrient contents. The trend of soil neutral protease is consistent with that of soil urease. However, soil acidic and alkaline proteases exhibited higher activities under the N and NPK treatments. These results suggested that under N and NPK treatments, where soils remained carbon-limited due to the absence of organic inputs, microorganisms appeared to rely more heavily on soil acid and alkaline proteases to decompose recalcitrant protein substrates and obtain N. In contrast, soil neutral protease activity increased substantially under M and MNPK treatments, reflecting the greater availability of labile carbon that supports the decomposition and release of labile organic nitrogen from organic fertilizer.

Soil phosphatase activity is an important indicator for assessing the direction and intensity of soil phosphorus biotransformation [60,61]. The organic fertilizer additions significantly enhanced the activities of soil acid, neutral, and alkaline phosphatases, consistent with previous studies [62,63]. The possible reasons include that the application of organic fertilizer introduces large amounts of labile organic carbon and organic phosphorus, thereby increasing microbial biomass and metabolic activity and supplying hydrolyzable organic-P substrates [64,65]. In addition, the increases in faba bean root biomass and root exudation elevate both root-derived phosphatases and microbial extracellular phosphatases [66,67]. The significant positive correlations between soil phosphatases and SOM and other nutrient indicators indicated that the enrichment of SOM and multiple nutrients markedly enhances microbial and root metabolic activity and extracellular enzyme secretion, thereby increasing phosphatase activity and promoting the mineralization and turnover of organic phosphorus in soil.

### 4.2. Responses of N-Cycling Enzyme-Encoding Genes to Different Fertilization Treatments

In this study, the higher relative abundance of nitrogen fixation functional genes from MNPK treatment likely implied the incorporation of organic fertilizer substantially increased soil SOM and TC contents, thereby providing essential carbon sources and energy substrates for diazotrophic microorganisms. Correlation analysis also revealed that the abundance of nitrogen fixation genes was significantly positively correlated with SOM and TC, underscoring the pivotal role of carbon availability in supporting the nitrogen fixation process. Moreover, the combined application of organic and inorganic fertilizers can optimize the soil C:N ratio, which further promotes the proliferation and activity of nitrogen-fixing microorganisms [68,69].

Functional genes related to N assimilation, mineralization, anammox, nitrification, denitrification, and dissimilatory nitrate reduction showed most abundance under N-only treatment. Sole nitrogen fertilization often results in limited available carbon and phosphorus (C, P co-limitation) and a reduced rhizosphere carbon flow due to less root biomass and exudates, which substantially restricts microbial growth and energy metabolism. Under such nutrient-impoverished conditions, microorganisms rely more on inorganic nitrogen assimilation, organic nitrogen mineralization, and inorganic nitrogen transformation processes to maintain nitrogen acquisition and basic metabolic activity, thereby enriching the associated functional genes [70,71,72]. In contrast, multi-nutrient sufficiency and enhanced rhizosphere carbon inputs expand microbial metabolic niches and functional gene diversity, reducing the relative investment in inorganic nitrogen uptake and transformation pathways and thus lowering the proportional abundance of these genes, despite possible greater microbial biomass and activity [73,74,75,76]. This is also consistent with long-term fertilization studies showing that N-only treatment leads to substantially greater nitrogen losses and lower nitrogen use efficiency than balanced or manure-based fertilization systems via leaching as NO_3_^−^ and gas volatilization or emissions as NH_3_, N_2_O, and N_2_ [77,78,79]. Such elevated N-loss pressures under N-only conditions can further stimulate microbial nitrogen transformation processes, leading to higher abundances of nitrification, denitrification, anammox functional genes. Faba bean yield was negatively associated with N-assimilation, anammox, denitrification, and dissimilatory nitrate reduction genes, but positively with assimilatory nitrate reduction genes. These contrasting correlations indicate that nitrogen transformation pathways associated with microbial nutrient stress (e.g., assimilation, dissimilatory nitrate reduction, anammox, denitrification) dominate in low-fertility soils and coincide with reduced crop performance, whereas assimilatory nitrate reduction increases under fertile, carbon-enriched soils that better support plant nitrogen uptake and higher yields.

### 4.3. Responses of P-Cycling Enzyme-Encoding Genes to Different Fertilization Treatments

The relative abundance of polyphosphate degradation genes was lowest under the NPK treatment, consistent with previous findings [80,81]. This can be primarily attributed to the direct input of soluble phosphorus fertilizers in the NPK treatment, which effectively alleviated soil phosphorus limitation and substantially reduced the dependence on intracellular polyphosphate degradation pathways. In contrast, the N-only treatment necessitated the activation of microbial polyphosphate degradation to release phosphorus as a compensatory strategy to secure essential P for basic metabolic needs, thereby stimulating its expression [82,83]. Under the M and MNPK treatments, the highest biomass and yield indicate greater plant demand for bioavailable P, likely increasing reliance on microbial P-releasing processes. Therefore, polyphosphate degradation pathways are still necessary to supplement more phosphorus under organic fertilization treatments [84].

Microorganisms drive soil phosphorus cycling by secreting organic acids and phosphatases, which were responsible for solubilizing inorganic P and releasing P from organic matter, respectively [85,86,87]. The generation of extracellular enzymes requires substantial resources, including carbon and nutrient inputs for constructing the enzyme molecules, as well as considerable metabolic energy to support protein synthesis and subsequent secretion [31,88]. For organic P mineralization functional genes, the NPK treatment showed the highest relative abundance, followed by M and MNPK, and lowest under N treatment. Acid phosphatase is predominantly derived from plants and fungi, while bacteria are the primary producers of alkaline phosphatase [89]. Phosphatase-driven organic P mineralization accounts for nearly 90% of total organic P turnover in soils [90,91]. These results are also consistent with our measured soil phosphatase activities, especially alkaline phosphatase activity, which was lowest under the N treatment and significantly higher under NPK, M, and MNPK. Because extracellular enzymes are short-lived, microorganisms must obtain a sufficient return on their investment to sustain growth, particularly under resource-limited conditions [31]. The severe carbon and nutrient limitation under the N treatment restricts microbial metabolic, making the energy-intensive mineralization process less favorable and lowering organic P-mineralizing gene abundance. Although organic P-mineralizing genes were slightly more abundant under NPK than under organic-added treatments, organic fertilization supported greater soil microbial biomass and still resulted in significantly higher soil phosphatase activities. The functional genes involved in inorganic P solubilization exhibited the highest abundance under the N-only treatment, significantly exceeding the other treatments. These results may reflect that inorganic P solubilization microbes are favored under low-P conditions, whereas high available phosphorus due to long-term P fertilization suppresses their abundance and activity through negative feedback regulation [92]. The NPK treatment directly supplied P and stimulated crop growth and nutrient demand, whereas M and MNPK increased SOM and nutrient levels, inducing the expression of P uptake and transport genes. This upregulation subsequently promoted plant phosphorus uptake, transport and utilization, ultimately leading to a significant increase in both biomass and grain yield of faba bean [87,93].

## 5. Conclusions

Long-term fertilization markedly reshaped soil nutrient status, enzyme activities, and microbial functional potential. Compared with chemical fertilization, organic fertilization significantly improved SOM, total and available nutrients, and achieved the highest faba bean yield. Urease, phosphatases, and neutral protease were key enzymatic indicators positively associated with soil fertility and productivity. Long-term fertilization also altered the abundance and coordination of N- and P-cycling functional genes. Genes related to assimilatory nitrate reduction and phosphate transport were strongly linked to higher soil nutrient availability and yield, while those involved in denitrification and anammox exhibited negative associations, reflecting microbial functional differentiation under different nutrient regimes. Overall, balanced application of organic and inorganic fertilizers effectively enhanced microbial nutrient cycling and soil biochemical functioning, offering an effective pathway for improving soil fertility and sustaining crop production in long-term agroecosystems.

It should be noted that soil sampling was conducted during a single crop season to ensure consistency across treatments. Although this approach effectively reflects the cumulative effects of long-term fertilization, it may not capture potential seasonal variations in microbial activity and enzyme dynamics. Future studies involving multi-seasonal sampling would provide further insight into temporal fluctuations of microbial functions under long-term fertilization regimes. Moreover, although Tax4Fun2 effectively predicts microbial functional potential, it does not directly reflect the active gene expression or enzymatic activity. Therefore, the observed relationships between predicted functional genes and soil enzyme activities should be interpreted as indicative of potential associations. Future studies employing metagenomic or metatranscriptomic analyses would provide more direct evidence of microbial functional dynamics under long-term fertilization treatments.

## Figures and Tables

**Table 1:** Soil characteristics and faba bean yields under different fertilization treatments.

Soil Properties	Long-Term Fertilization Treatments			
	N	NPK	M	MNPK
pH	7.78 ± 0.05 a	7.67 ± 0.05 b	7.73 ± 0.01 a	7.53 ± 0.02 c
EC (μS/cm)	343.38 ± 114.81 b	432.06 ± 77.81 b	412.23 ± 40.89 b	650.13 ± 84.31 a
TN (g/kg)	1.43 ± 0.12 b	1.47 ± 0.08 b	2.60 ± 0.17 a	2.56 ± 0.66 a
TP (g/kg)	0.61 ± 0.03 b	0.47 ± 0.17 b	2.95 ± 0.67 a	2.62 ± 0.67 a
TC (g/kg)	16.14 ± 0.42 b	17.68 ± 0.68 b	27.81 ± 1.99 a	28.82 ± 1.99 a
SOM (g/kg)	19.54 ± 2.73 b	22.08 ± 1.41 b	39.16 ± 3.36 a	43.40 ± 4.62 a
AP (mg/kg)	21.70 ± 3.71 b	28.40 ± 4.88 b	208.25 ± 10.12 a	228.28 ± 32.28 a
AK (mg/kg)	28.03 ± 4.11 c	68.16 ± 5.01 c	329.44 ± 60.86 b	480.27 ± 98.39 a
NO_3_^−^-N (mg/kg)	6.21 ± 0.63 a	5.25 ± 0.59 a	4.91 ± 1.22 a	5.45 ± 1.45 a
NH_4_^+^-N (mg/kg)	156.81 ± 45.32 a	148.66 ± 26.17 a	152.99 ± 26.40 a	168.80 ± 25.68 a
GY (kg/ha)	122.32 ± 27.87 c	1464.58 ± 306.72 b	2563.99 ± 186.09 a	2823.51 ± 366.67 a

Data are means ± SD (n = 4). Different lowercase letters at the end of each value mean significant differences among treatments in the same row (*p* < 0.05, Duncan’s test). Abbreviations: N only nitrogen fertilizer, NPK nitrogen, phosphorus and potassium fertilizers, M only organic fertilizer, MNPK NPK + M. EC, electrical conductivity; AK, available potassium; AP, available phosphorus; SOM, soil organic matter; TC, total carbon; TN, total nitrogen; TP, total phosphorus; GY, grain yield.

## Data Availability

The original data presented in the study are openly available in NCBI Sequence Read Archive database with the accession number PRJNA1118028.

## Abbreviations

The following abbreviations are used in this manuscript:

N	nitrogen
P	phosphorus
K	potassium
NPK	combined N, P, and K fertilization
M	organic fertilizer alone
MNPK	NPK plus organic fertilizer
EC	electrical conductivity
TN	total nitrogen
TP	total phosphorus
TC	total carbon
SOM	soil organic matter
AP	available phosphorus
AK	available potassium
GY	grain yield
Urease	soil urease
ACPro	soil acid protease
NEPro	soil neutral protease
ALPro	soil alkaline protease
ACP	soil acid phosphatase
NEP	soil neutral phosphatase
ALP	soil alkaline phosphatase

## Appendix A

**Table A1:** Soil bacterial KEGG pathways that related to nitrogen and phosphorus cycles.

Nutrient Cycle	Function	KEGG Pathway	Corresponding Gene	Description
Nitrogen	Anammox	K00368	*nirK*	nitrite reductase (NO-forming) [EC:1.7.2.1]
		K01428	*ureC*	urease subunit alpha [EC:3.5.1.5]
		K01429	*ureB*	urease subunit beta [EC:3.5.1.5]
		K01430	*ureA*	urease subunit gamma [EC:3.5.1.5]
		K10535	*hao*	hydroxylamine dehydrogenase [EC:1.7.2.6]
	N fixation	K00531	*anfG*	nitrogenase delta subunit [EC:1.18.6.1]
		K02586	*nifD*	nitrogenase molybdenum-iron protein alpha chain [EC:1.18.6.1]
		K02588	*nifH*	nitrogenase iron protein NifH [EC:1.18.6.1]
		K02591	*nifK*	nitrogenase molybdenum-iron protein beta chain [EC:1.18.6.1]
		K02592	*nifN*	nitrogenase molybdenum-iron protein NifN
		K02593	*nifT*	nitrogen fixation protein NifT
		K02594	*nifV*	homocitrate synthase NifV [EC:2.3.3.14]
		K02595	*nifW*	nitrogenase-stabilizing/protective protein
		K02596	*nifX*	nitrogen fixation protein NifX
	N mineralization	K00260	*gudB*	glutamate dehydrogenase [EC:1.4.1.2]
		K00261	*GLUD1_2*	glutamate dehydrogenase (NAD(P)+) [EC:1.4.1.3]
		K00262	*gdhA*	glutamate dehydrogenase (NADP+) [EC:1.4.1.4]
	N assimilation	K00265	*gltB*	glutamate synthase (NADPH/NADH) large chain [EC:1.4.1.13 1.4.1.14]
		K00266	*gltD*	glutamate synthase (NADPH/NADH) small chain [EC:1.4.1.13 1.4.1.14]
		K00284	*GLU*	glutamate synthase (ferredoxin) [EC:1.4.7.1]
		K00459	*nmo*	nitronate monooxygenase [EC:1.13.12.16]
		K01424	*ansB*	L-asparaginase [EC:3.5.1.1]
		K01425	*glsA*	glutaminase [EC:3.5.1.2]
		K01953	*asnB*	asparagine synthase (glutamine-hydrolysing) [EC:6.3.5.4]
	Nitrification	K10535	*hao*	hydroxylamine dehydrogenase [EC:1.7.2.6]
		K10944	*pmoA-amoA*	methane/ammonia monooxygenase subunit A [EC:1.14.18.3 1.14.99.39]
		K10945	*pmoB-amoB*	methane/ammonia monooxygenase subunit B
		K10946	*pmoC-amoC*	methane/ammonia monooxygenase subunit C
	Denitrification	K00368	*nirK*	nitrite reductase (NO-forming) [EC:1.7.2.1]
		K00370	*narG*, *narZ*, *nxrA*	nitrate reductase/nitrite oxidoreductase, alpha subunit [EC:1.7.5.1 1.7.99.-]
		K00371	*narH*, *narY*, *nxrB*	nitrate reductase/nitrite oxidoreductase, beta subunit [EC:1.7.5.1 1.7.99.-]
		K00374	*narI, narV*	nitrate reductase gamma subunit [EC:1.7.5.1 1.7.99.-]
		K00376	*nosZ*	nitrous-oxide reductase [EC:1.7.2.4]
		K02305	*norC*	nitric oxide reductase subunit C
		K02567	*napA*	periplasmic nitrate reductase NapA [EC:1.7.99.-]
		K02568	*napB*	cytochrome c-type protein NapB
		K04561	*norB*	nitric oxide reductase subunit B [EC:1.7.2.5]
		K15864	*nirS*	nitrite reductase (NO-forming)/hydroxylamine reductase [EC:1.7.2.1 1.7.99.1]
	Assimilatory nitrate reduction	K00265	*gltB*	glutamate synthase (NADPH/NADH) large chain [EC:1.4.1.13 1.4.1.14]
		K00360	*nasB*	assimilatory nitrate reductase electron transfer subunit [EC:1.7.99.-]
		K00366	*nirA*	ferredoxin-nitrite reductase [EC:1.7.7.1]
		K00367	*narB*	ferredoxin-nitrate reductase [EC:1.7.7.2]
		K00372	*nasA*	assimilatory nitrate reductase catalytic subunit [EC:1.7.99.-]
		K01915	*glnA*, *GLUL*	glutamine synthetase [EC:6.3.1.2]
		K02575	*NRT2*, *narK*, *nrtP*, *nasA*	MFS transporter, NNP family, nitrate/nitrite transporter
		K15577	*nrtB*, *nasE*, *cynB*	nitrate/nitrite transport system permease protein
		K15578	*nrtC, nasD*	nitrate/nitrite transport system ATP-binding protein [EC:3.6.3.-]
		K15579	*narC*	nitrate/nitrite transport system ATP-binding protein
	Dissimilatory nitrate reduction	K00362	*nirB*	nitrite reductase (NADH) large subunit [EC:1.7.1.15]
		K00363	*nirD*	nitrite reductase (NADH) small subunit [EC:1.7.1.15]
		K00370	*narG*, *narZ*, *nxrA*	nitrate reductase/nitrite oxidoreductase, alpha subunit [EC:1.7.5.1 1.7.99.-]
		K00371	*narH*, *narY*, *nxrB*	nitrate reductase/nitrite oxidoreductase, beta subunit [EC:1.7.5.1 1.7.99.-]
		K00373	*narJ*, *narW*	nitrate reductase molybdenum cofactor assembly chaperone NarJ/NarW
		K00374	*narI*, *narV*	nitrate reductase gamma subunit [EC:1.7.5.1 1.7.99.-]
		K02567	*napA*	periplasmic nitrate reductase NapA [EC:1.7.99.-]
		K02568	*napB*	cytochrome c-type protein NapB
		K02569	*napC*	cytochrome c-type protein NapC
		K03385	*nrfA*	nitrite reductase (cytochrome c-552) [EC:1.7.2.2]
		K04013	*nrfB*	cytochrome c-type protein NrfB
		K04014	*nrfC*	protein NrfC
		K04015	*nrfD*	protein NrfD
		K15576	*nrtA*, *nasF*, *cynA*	nitrate/nitrite transport system substrate-binding protein
		K15876	*nrfH*	cytochrome c nitrite reductase small subunit
Phosphorus	Polyphosphate polymerization	K00937	*ppk1*	polyphosphate kinase [EC:2.7.4.1]
		K15986	*ppaC*	manganese-dependent inorganic pyrophosphatase [EC:3.6.1.1]
	Polyphosphate degradation	K00858	*ppnK*, *NADK*	NAD+ kinase [EC:2.7.1.23]
		K00873	*PK*, *pyk*	pyruvate kinase [EC:2.7.1.40]
		K00886	*ppgK*	polyphosphate glucokinase [EC:2.7.1.63]
		K00940	*ndk*, *NME*	nucleoside-diphosphate kinase [EC:2.7.4.6]
		K00951	*relA*	GTP pyrophosphokinase [EC:2.7.6.5]
		K01139	*spoT*	GTP diphosphokinase/guanosine-3,5-bis(diphosphate) 3-diphosphatase [EC:2.7.6.5 3.1.7.2]
		K03787	*surE*	5-nucleotidase [EC:3.1.3.5]
		K21138	*HDDC3*	guanosine-3,5-bis(diphosphate) 3-pyrophosphohydrolase [EC:3.1.7.2]
		K22468	*ppk2*	polyphosphate kinase [EC:2.7.4.1]
	P starvation response regulation	K02039	*phoU*	phosphate transport system protein
		K07636	*phoR*	two-component system, OmpR family, phosphate regulon sensor histidine kinase PhoR [EC:2.7.13.3]
		K07657	*phoB*	two-component system, OmpR family, phosphate regulon response regulator PhoB
		K07658	*phoP*	two-component system, OmpR family, alkaline phosphatase synthesis response regulator PhoP
		K10916	*cqsS*	two-component system, CAI-1 autoinducer sensor kinase/phosphatase CqsS [EC:2.7.13.3 3.1.3.-]
	P uptake and transport system	K02036	*pstB*	phosphate transport system ATP-binding protein [EC:3.6.3.27]
		K02037	*pstB*	phosphate transport system permease protein
		K02038	*pstA*	phosphate transport system permease protein
		K02040	*pstS*	phosphate transport system substrate-binding protein
		K02041	*phnC*	phosphonate transport system ATP-binding protein [EC:3.6.3.28]
		K02042	*phnE*	phosphonate transport system permease protein
		K02043	*phnF*	GntR family transcriptional regulator, phosphonate transport system regulatory protein
		K02044	*phnD*	phosphonate transport system substrate-binding protein
		K02440	*GLPF*	glycerol uptake facilitator protein
		K02443	*glpP*	glycerol uptake operon antiterminator
		K02444	*glpR*	DeoR family transcriptional regulator, glycerol-3-phosphate regulon repressor
		K02445	*glpT*	MFS transporter, OPA family, glycerol-3-phosphate transporter
		K02757	*bglF*	PTS system, beta-glucoside-specific IIC component
		K03306	*pit*	inorganic phosphate transporter, PiT family
		K03324	*yjbB*	phosphate:Na+ symporter
		K05781	*phnK*	putative phosphonate transport system ATP-binding protein
		K05813	*ugpB*	sn-glycerol 3-phosphate transport system substrate-binding protein
		K05814	*ugpA*	sn-glycerol 3-phosphate transport system permease protein
		K05815	*ugpE*	sn-glycerol 3-phosphate transport system permease protein
		K05833	*K05833*	putative ABC transport system ATP-binding protein
		K07220	*K07220*	uncharacterized protein
		K07221	*oprO_P*	phosphate-selective porin OprO and OprP
		K11082	*phnV*	2-aminoethylphosphonate transport system permease protein
		K16322	*pit*	low-affinity inorganic phosphate transporter
	Inorganic P solubilization	K00112	*glpB*	glycerol-3-phosphate dehydrogenase subunit B [EC:1.1.5.3]
		K00113	*glpC*	glycerol-3-phosphate dehydrogenase subunit C [EC:1.1.5.3]
		K00117	*gcd*	quinoprotein glucose dehydrogenase [EC:1.1.5.2]
		K01507	*ppa*	inorganic pyrophosphatase [EC:3.6.1.1]
		K01524	*ppx*	exopolyphosphatase/guanosine-5-triphosphate,3-diphosphate pyrophosphatase [EC:3.6.1.11 3.6.1.40]
		K06136	*pqqB*	pyrroloquinoline quinone biosynthesis protein B
		K06137	*pqqC*	pyrroloquinoline-quinone synthase [EC:1.3.3.11]
		K06138	*pqqD*	pyrroloquinoline quinone biosynthesis protein D
		K06139	*pqqE*	pyrroloquinoline quinone biosynthesis protein E
	Organic P mineralization	K00105	*E1.1.3.21*	alpha-glycerophosphate oxidase [EC:1.1.3.21]
		K00864	*glpK*	glycerol kinase [EC:2.7.1.30]
		K00906	*aceK*	isocitrate dehydrogenase kinase/phosphatase [EC:2.7.11.5 3.1.3.-]
		K01077	*E3.1.3.1*, *phoA*, *phoB*	alkaline phosphatase [EC:3.1.3.1]
		K01079	*serB*	phosphoserine phosphatase [EC:3.1.3.3]
		K01083	*E3.1.3.8*	3-phytase [EC:3.1.3.8]
		K01091	*gph*	phosphoglycolate phosphatase [EC:3.1.3.18]
		K01092	*IMPA*	myo-inositol-1(or 4)-monophosphatase [EC:3.1.3.25]
		K01093	*appA*	4-phytase/acid phosphatase [EC:3.1.3.26 3.1.3.2]
		K01113	*phoD*	alkaline phosphatase D [EC:3.1.3.1]
		K01126	*E3.1.4.46*, *glpQ*, *ugpQ*	glycerophosphoryl diester phosphodiesterase [EC:3.1.4.46]
		K01841	*pepM*	phosphoenolpyruvate phosphomutase [EC:5.4.2.9]
		K02043	*phnF*	GntR family transcriptional regulator, phosphonate transport system regulatory protein
		K02203	*thrH*	phosphoserine/homoserine phosphotransferase [EC:3.1.3.3 2.7.1.39]
		K03270	*kdsC*	3-deoxy-D-manno-octulosonate 8-phosphate phosphatase (KDO 8-P phosphatase) [EC:3.1.3.45]
		K03430	*phnW*	2-aminoethylphosphonate-pyruvate transaminase [EC:2.6.1.37]
		K03788	*aphA*	acid phosphatase (class B) [EC:3.1.3.2]
		K05306	*phnX*	phosphonoacetaldehyde hydrolase [EC:3.11.1.1]
		K05518	*rsbX*	phosphoserine phosphatase RsbX [EC:3.1.3.3]
		K05774	*phnN*	ribose 1,5-bisphosphokinase [EC:2.7.4.23]
		K05780	*phnL*	alpha-D-ribose 1-methylphosphonate 5-triphosphate synthase subunit PhnL [EC:2.7.8.37]
		K05816	*ugpC*	sn-glycerol 3-phosphate transport system ATP-binding protein [EC:3.6.3.20]
		K06162	*phnM*	alpha-D-ribose 1-methylphosphonate 5-triphosphate diphosphatase [EC:3.6.1.63]
		K06163	*phnJ*	alpha-D-ribose 1-methylphosphonate 5-phosphate C-P lyase [EC:4.7.1.1]
		K06164	*phnI*	alpha-D-ribose 1-methylphosphonate 5-triphosphate synthase subunit PhnI [EC:2.7.8.37]
		K06165	*phnH*	alpha-D-ribose 1-methylphosphonate 5-triphosphate synthase subunit PhnH [EC:2.7.8.37]
		K06166	*phnG*	alpha-D-ribose 1-methylphosphonate 5-triphosphate synthase subunit PhnG [EC:2.7.8.37]
		K06167	*phnP*	phosphoribosyl 1,2-cyclic phosphate phosphodiesterase [EC:3.1.4.55]
		K06193	*phnA*	protein PhnA
		K07048	*aceK*	phosphotriesterase-related protein
		K07175	*PhoH2*	PhoH-like ATPase
		K07315	*rsbU_P*	phosphoserine phosphatase RsbU/P [EC:3.1.3.3]
		K08483	*ptsI*	phosphotransferase system, enzyme I, PtsI [EC:2.7.3.9]
		K08484	*ptsP*	phosphotransferase system, enzyme I, PtsP [EC:2.7.3.9]
		K09474	*phoN*	acid phosphatase (class A) [EC:3.1.3.2]
		K09994	*phnO*	aminoalkylphosphonate N-acetyltransferase [EC:2.3.1.-]
		K15781	*serB-plsC*	putative phosphoserine phosphatase/1-acylglycerol-3-phosphate O-acyltransferase [EC:3.1.3.3 2.3.1.51]
		K16055	*TPS*	trehalose 6-phosphate synthase/phosphatase [EC:2.4.1.15 3.1.3.12]
